# ClicO FS: an interactive web-based service of Circos

**DOI:** 10.1093/bioinformatics/btv433

**Published:** 2015-07-29

**Authors:** Wei-Hien Cheong, Yung-Chie Tan, Soon-Joo Yap, Kee-Peng Ng

**Affiliations:** ^1^Department of Science and Technology, Codon Genomics S/B, Taman Dutamas, Balakong, 43200 Seri Kembangan, Malaysia and; ^2^Department of Medical Microbiology, Faculty of Medicine, University of Malaya, Kuala Lumpur, Malaysia

## Abstract

**Summary**: We present ClicO Free Service, an online web-service based on Circos, which provides a user-friendly, interactive web-based interface with configurable features to generate Circos circular plots.

**Availability and implementation**: Online web-service is freely available at http://clicofs.codoncloud.com

**Contact**: soonjoo.yap@codongenomics.com

**Supplementary information:** Supplementary data are available at *Bioinformatics* online.

## 1 Introduction

Circos (Krzywinski *et al.*, 2009) is a Perl language-based tool to represent visual data in a circular form. The native Circos software is provided through a command-line interface (CLI). Installation and configuration of the software, though not difficult for users with command line experience, pose a certain degree of difficulty to those who are not familiar with the CLI.

There are other alternatives to Circos that enable plotting of circular images, which including Circoletto, CIRCUS and RCircos (Darzentas, 2010; Naquin *et al.*, 2014; Zhang *et al.*, 2013). Circoletto and CIRCUS have been developed for more specialised functions. The former integrates Basic Local Alignment Search Tool or BLAST (Altschul *et al.*, 1990) results and the Circos plot via a web-based user interface (UI), whereas the latter specializes in plotting structure variants of genomes. They are not flexible in generating plots with different data types. On the other hand, RCircos supports Circos data track plotting, but it is based on the R-package CLI.

Some of the applications above require setup, configuration and coding skills in order to fully utilize the applications. Users with little or no experience in the CLI environment could encounter a demanding learning curve. To address the issue, ClicO FS (Open Circular Layout Interactive Converter) is developed. ClicO FS is a user-friendly web service which allows users to generate Circos plots easily. Not all functions of Circos can be done by ClicO FS, such as chromosome axis breaks.

## 2 Methods

ClicO FS is built using Ruby on Rails as the back-end technology, and Twitter Bootstrap as the front-end framework. ClicO FS receives the required files through user upload. Three types of files are required by Circos, namely (i) karyotype, (ii) data and (iii) configuration files, while ClicO FS requires only the first two file types. The karyotype file defines the axes, which are typically chromosomes, sequence contigs, or clones in the biological context. It is a 7-column format file containing information for each axis, including unique identifier, label, size and color. The data files are another input files from which to draw various types of data. Data types currently supported by ClicO FS includes links, histograms, line plots, scatter plots, heatmaps, tiles, texts, connectors and highlights. The data file formats are simple, generally involving three to seven columns depending on the data track to be drawn. Detailed descriptions on the file formats are specified on the ClicO FS interface. All files accepted by ClicO FS are of the same format as Circos to facilitate ease the use when switching between the two programmes.

ClicO FS does not require users to upload configuration files. All configurations can be set in the user interface. As ClicO FS is designed with user-friendliness as a priority, default parameters that are not configurable are given to some less commonly used settings. However, advanced users are allowed to input more configurations like backgrounds, rules, axes, label snuggling and highlights, in a special text box in ClicO FS.

Some additional functions are provided, such as the assignment of various color schemes to karyotypes. Besides, users can preview the output at any time while testing the configurations, without saving them.

Registered ClicO FS users can create multiple independent projects. All associated data and configurations are stored in databases dedicated to the respective projects. Thus, users can retrieve or continue to work on the project the next time they log in.

In addition to that, ClicO FS provides a pre-configured, template-based approach that can speed up the generation of some common forms of presentation. For instance, GenBank database is one of the most common sources of sequence data. Users can upload GenBank format files to ClicO FS. With just five clicks, information like genes, GC contents and structural RNAs of that genome can be visualized.

Functionality of Circos is diverse and dynamic. To expand the functionality of ClicO FS in line with that of Circos. To address this issue, users of ClicO FS are allowed to download all configuration and data files of the project that they creat. These files can be used as a template, to add elements not supported by ClicO FS, and run using Circos from a terminal.

## 3 Results

**Fig. 1.**
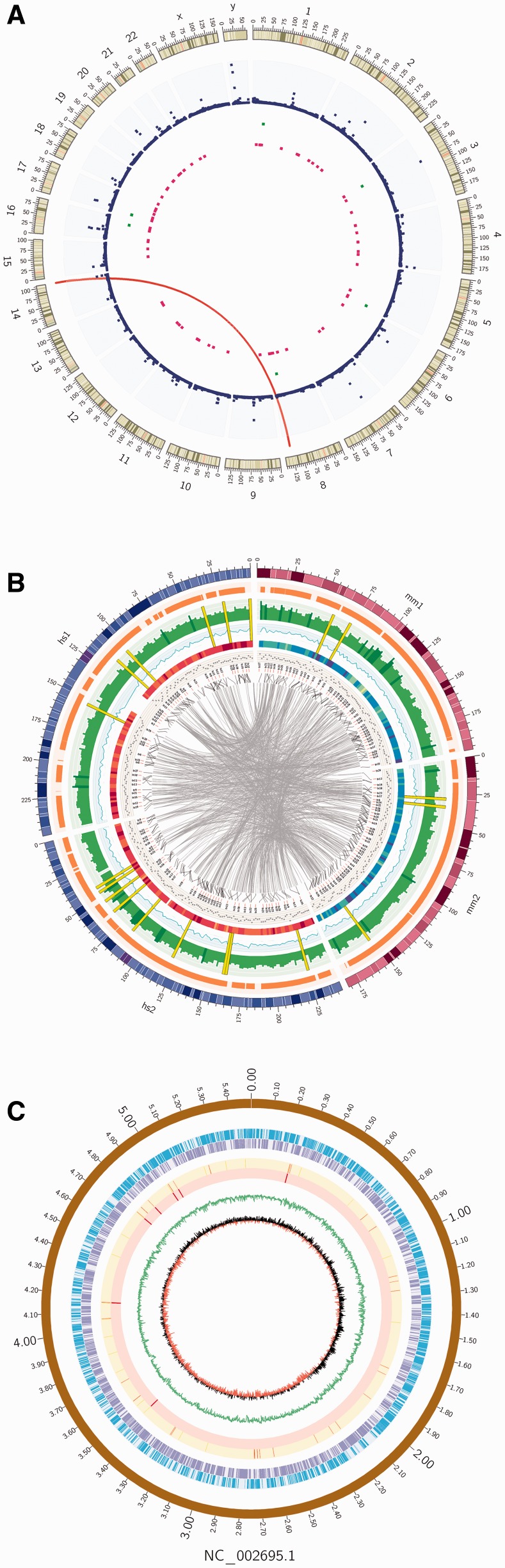
ClicO FS Plots. Re-produced circular plots based on publication by Love *et al.* (2012) (**A**) Comparative genomics of human and mouse using simulated data show that ClicO FS is capable to generate various data types including tiles, histogram, line plot, heatmap, scatter plot, text, connector, links and highlight plots (**B**). Circular plots of *Escherichia coli* O157:H7 genome (**C**). The tracks from outer to inner rings after the scale (in Mb) represent chromosome, forward genes, reverse genes, tRNA, rRNA, GC plot and GC skew

To demonstrate the capability of ClicO FS to generate Circos images without CLI interaction, attempts were made to reproduce relevant published images using ClicO FS. A randomly selected publication with different data types, including links and scatter plots was chosen (Love *et al.*, 2012). Data was extracted from the published paper, and was converted according to the ClicO FS input format. Then ensuing reproduced plot is similar to the original Circos plot from the paper (Fig. 1A and Supplement Data) with the pattern and the meaning of the data preserved.

In addition, simulated data was generated to plot human and mouse genomes with ClicO FS. Figure 1B shows that ClicO FS is able to generate links, histogram, line plot, scatter plot, heatmap, tiles, text, connector and highlight. Moreover, background color, axes and rules were applied on histogram successfully using the advanced text box.

To test the template-based plotting function of ClicO FS, a randomly selected genbank file of a bacterium genome *Escherichia coli* O157:H7 (NC_002695) was used. Information on the genome including genes, GC plots, and structural RNAs was successfully plotted by ClicO FS (Fig. 1C).

## 4 Conclusions and outlook

ClicO FS was developed to provide a user friendly interface to Circos. It allows researchers to access the powerful Circos application while reducing the complexity required in setting up the application as well as reducing the time required to generate a Circos plot. ClicO FS provides an easy-to-understand workflow with step by step guide for the users to input their data and configure the image output. Currently, ClicO FS is being improved to support some specific applications such as plotting BLAST data for comparative genomics, genetic linkage map data and transcriptome analysis data. These applications will be developed as plugins to simplify the process of generating circular image. We will continue to develop ClicO FS to provide more features and support to the research community.

## Funding

This work was supported by a High Impact Research (HIR) grant from the Ministry of Education Malaysia [UM.C/625/1/HIR/MOHE/MED/31 (Account no. H20001-00-E000070)] and Codon Genomics Sdn. Bhd. We thank reviewers for their constructive comments.

*Conflicts of Interest:* none declared.

## Supplementary Material

Supplementary Data
