# Supplementary material for: ClicO FS: an interactive web-based service of Circos
Source: Bioinformatics. 2015 Jul 29;31(22):3685–7. doi: 10.1093/bioinformatics/btv433 (PMC4817113; doi:10.1093/bioinformatics/btv433)
Supplement: Supplementary Data [file supp_31_22_3685__index.html]

ClicO FS: An interactive web-based service of Circos — ClicO FS: an interactive web-based service of Circos — ClicO FS: an interactive web-based service of Circos — Supplementary Data 

# ClicO FS: an interactive web-based service of Circos

## Supplementary Data

files

- Supplementary Data - doc file
