# Supplementary material for: ClicO FS: an interactive web-based service of Circos
Source: Bioinformatics. 2015 Jul 29;31(22):3685–7. doi: 10.1093/bioinformatics/btv433 (PMC4817113; doi:10.1093/bioinformatics/btv433)
Supplement: Supplementary Data [file supp_btv433_ClicOFSSupplementaryFile.doc]

**Supplementary File**


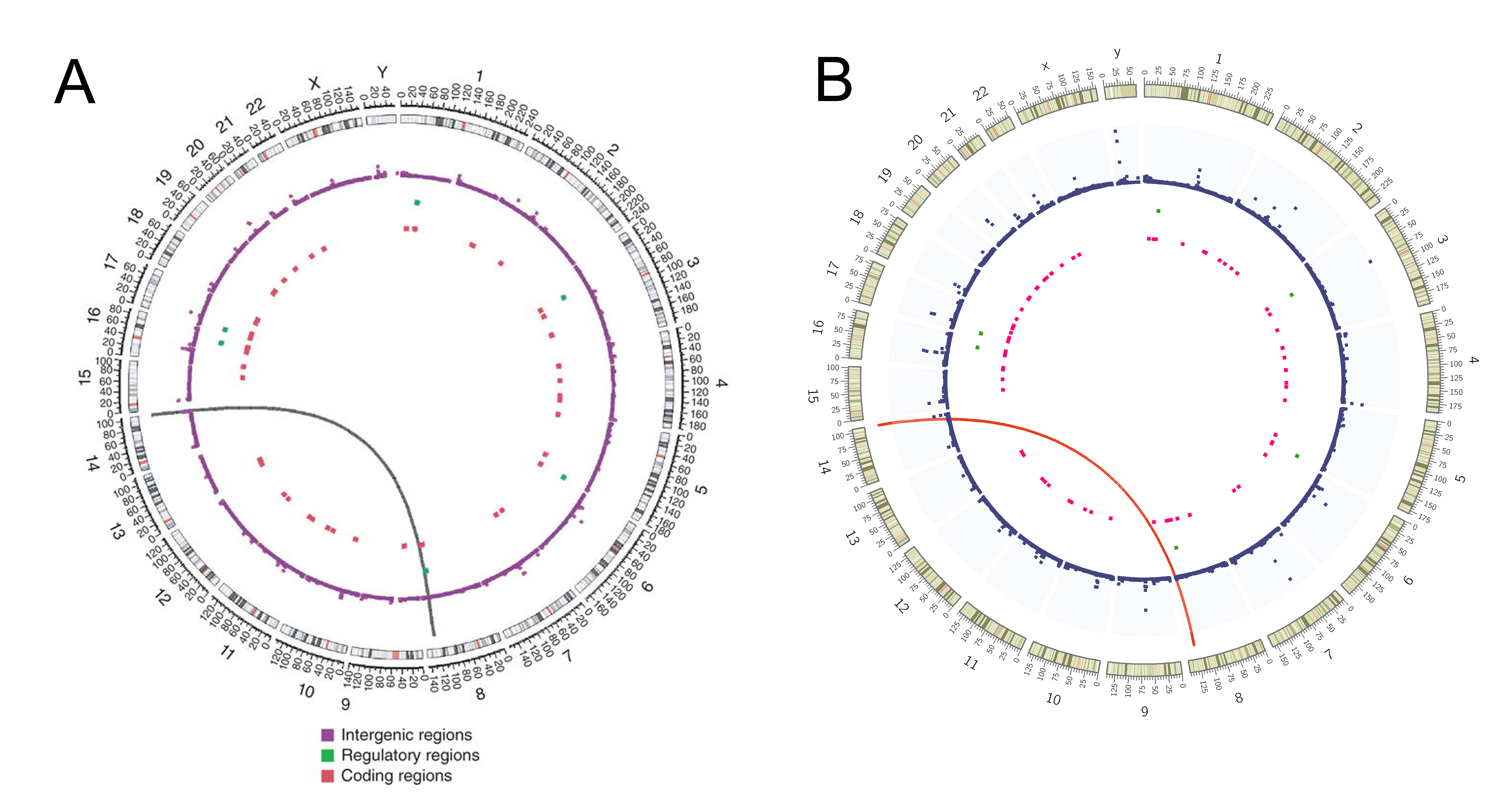


**Supplementary Fig. 1**. Original plot from Love *et al*. (2012) and reproduced circular plots by ClicO FS (A and B, respectively).
